# Isoform-Directed Control of c-Myc Functions: Understanding the Balance from Proliferation to Growth Arrest

**DOI:** 10.3390/ijms242417524

**Published:** 2023-12-15

**Authors:** Agata Kubickova, Juan Bautista De Sanctis, Marian Hajduch

**Affiliations:** 1 Institute of Molecular and Translational Medicine, Palacky University and University Hospital Olomouc, Hnevotinska 1333/5, 77900 Olomouc, Czech Republic; agata.kubickova@upol.cz (A.K.); juanbautista.desanctis@upol.cz (J.B.D.S.); 2 Institute of Molecular and Translational Medicine, Czech Advanced Technology and Research Institute, Palacky University in Olomouc, Hnevotinska 1333/5, 77900 Olomouc, Czech Republic

**Keywords:** c-Myc locus structure, p64 isoform, p67 isoform, c-Myc S, MycHex1, mrtl

## Abstract

The transcription factor c-Myc, a key regulator of cellular processes, has long been associated with roles in cell proliferation and apoptosis. This review analyses the multiple functions of c-Myc by examining the different c-Myc isoforms in detail. The impact of different c-Myc isoforms, in particular p64 and p67, on fundamental biological processes remains controversial. It is necessary to investigate the different isoforms in the context of proto-oncogenesis. The current knowledge base suggests that neoplastic lesions may possess the means for self-destruction via increased c-Myc activity. This review presents the most relevant information on the c-Myc locus and focuses on a number of isoforms, including p64 and p67. This compilation provides a basis for the development of therapeutic approaches that target the potent growth arresting and pro-apoptotic functions of c-Myc. This information can then be used to develop targeted interventions against specific isoforms with the aim of shifting the oncogenic effects of c-Myc from pro-proliferative to pro-apoptotic. The research summarised in this review can deepen our understanding of how c-Myc activity contributes to different cellular responses, which will be crucial in developing effective therapeutic strategies; for example, isoform-specific approaches may allow for precise modulation of c-Myc function.

## 1. Introduction

The c-Myc transcription factor holds a central role in molecular biology and cancer research due to the orchestration of various cellular processes through intricate control of gene expression [1]. Structurally, c-Myc is a nuclear protein that forms heterodimers with the Max (MYC Associated Factor X) protein. This complex binds to specific DNA sequences, known as E-boxes, in gene promoters [2]. In terms of function, c-Myc either activates or represses the transcription of genes that are critical to cell growth, proliferation, and apoptosis. As such, it has a significant influence on the fate of cells. When regulating cell growth, c-Myc upregulates the transcription of genes encoding protein products involved in the cell cycle (such as E2Fs transcription factors, cyclins, and cyclin-dependent kinases) and suppresses the transcription of genes linked with the inhibition of mitogenic processes (for instance cyclin-dependent kinase inhibitors p21, p27) [3,4,5,6,7]. Hence, it is clear that dysregulated c-Myc activity can lead to unbridled cell proliferation [8]. This pro-proliferative effect is central to the involvement of c-Myc in tumourigenesis.

The dysregulation of c-Myc is a common hallmark of cancer [9]. Many cancer cells exhibit elevated levels of c-Myc, contributing to uncontrolled growth and the propensity to form tumours. Alterations in the c-Myc gene, such as mutations or amplifications, are frequently observed in various cancers, including breast, lung, and colorectal [10,11,12]. The aberrant expression of c-Myc can be triggered by many factors, including mitogenic signals, growth factors, and cellular stress responses; this highlights how a complex interplay of regulatory mechanisms is involved in the control of c-Myc [2].

In addition to stimulating cell growth, c-Myc is also involved in apoptosis, or the process of programmed cell death [13,14]. This dual role adds a further level of complexity to how c-Myc contributes to tumourigenesis. Under certain conditions, c-Myc can promote cell survival by upregulating anti-apoptotic genes, which can block pro-apoptotic signals in cancer cells [15]. However, c-Myc can also induce apoptosis when required to safeguard against the uncontrolled proliferation of damaged cells. These paradoxical roles underscore how critical c-Myc is to maintaining a healthy balance within each cell.

In addition to involvement in cancer, c-Myc also plays a pivotal role in stem cell regulation [16]. More specifically, c-Myc is instrumental in maintaining the pluripotency of embryonic stem cells, which ensures that these cells can differentiate into various cell types; this includes erythroid precursors, which demonstrate a decreased potential for differentiation in certain inherited diseases, such as Diamond-Blackfan anaemia [17,18]. Additionally, c-Myc is crucial in the reprogramming of somatic cells into induced pluripotent stem cells (iPSCs), a role which is highly relevant for regenerative medicine and tissue engineering [19].

Establishing that c-Myc plays a pivotal role in tumourigenesis has led researchers to focus on developing therapies that target this transcription factor [20]. The prevailing avenue of research is exploring how to selectively inhibit the c-Myc activity in cancer cells to disrupt uncontrolled proliferation, which holds immense promise for how cancer is treated.

c-Myc regulates a wide array of cellular processes, ranging from cell growth to programmed cell death [21]. Influences on both uncontrolled cell growth and apoptosis underscore why c-Myc is highly relevant to cancer research. The ongoing quest to unravel the complex pathways and conditions that affect c-Myc function may uncover the basis for how to develop targeted therapies for more effective cancer treatment.

The regulation and functioning of c-Myc significantly impacts the phenotype of a cell. However, despite intensive study, the mechanisms through which proteins encoded by the c-Myc locus are involved in diverse cellular processes remain poorly understood [22,23,24]. The very diverse functions of this transcription factor may be due to the complex and understudied polycistronic locus. A closer understanding of the structure and function of the individual c-Myc isoforms may be a key in the development of safe targeted therapies against different c-Myc dependent tumour types.

**Figure 1:**
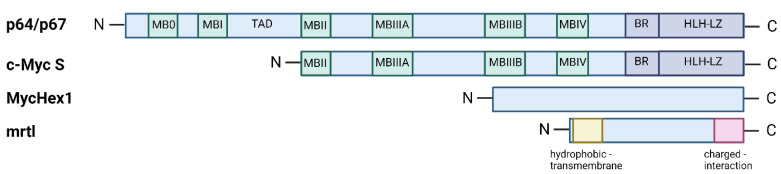
Schematic representation of the protein structures encoded by the c-Myc locus. The N- and C- labels represent the N-terminal and C-terminal regions of the proteins, respectively. The full-length protein structures are indicated by light blue rectangles. Conserved regions of the Myc boxes are labelled MB0 to MBIV and visualised by light green rectangles. The transcriptional activation domain (TAD) is located between MBI and MBII of p64 and p67. The dimerisation domains of p64, p67, and c-Myc S are shown in dark blue and are divided into the basic region (BR) and the helix-loop-helix leucine zipper domain (HLH-LZ). In the case of mrtl, the hydrophobic region is shown in light yellow and the charged region is represented by a light red rectangle. The structure of MycHex1 has not yet been characterised in detail. Created with BioRender.com.

The c-Myc proteins share many features with other transcriptional regulators. These proteins are localised to the nucleus, can be phosphorylated, and have relatively short half-lives, all characteristics which suggest that these proteins are regulated at various levels [25,26]. Specific molecular functions have been assigned to the C- and N-terminal regions of c-Myc-encoded proteins. For instance, the C-terminal domain of c-Myc proteins shares structural similarities with members of the basic helix-loop-helix leucine zipper (bHLH-LZ) superfamily of transcription factors [23] (Figure 1). The dimerisation of c-Myc with Max, a member of the bHLH-LZ family [27], through the HLH-LZ region of both proteins, facilitates sequence-specific binding to the CACGTG motif or E-box Myc site (EMS) in DNA [28,29]. The c-Myc proteins can stimulate transcription by binding to EMS sequences, whereas an excess of Max antagonises this transcriptional activity in cells [30,31,32]. Activation of transcription by the c-Myc proteins also requires intact N- and C- domains. The N-terminal region of the c-Myc proteins functions as a transactivation domain, and deletions of highly conserved regions within the N-terminal domain, called Myc boxes, reduce its transactivation function [32,33] (Figure 1). In addition, transactivation can be modulated by proteins that interact with the N-terminal domain of c-Myc proteins, including the TATA-binding protein (TBP) and the pRb-like protein p107 [34,35].

c-Myc primarily functions in the nucleus as a transcription factor for three RNA polymerases. The c-Myc/Max heterodimer, via the activation or repression of pol II target genes, is critical in progression to the cell cycle from quiescence [2,36,37,38,39,40]. c-Myc significantly impacts the overall rate of intracellular protein synthesis by stimulating the activity of pol I (rRNA synthesis) and pol III, which are involved in the production of many components of the translational apparatus (e.g., translation initiation factors, ribosomal proteins) [41,42,43,44,45]. As such, c-Myc^−/−^ cells have been found to show reduced RNA and protein synthesis rates, along with prolonged cell division [46].

The ability to induce growth arrest and apoptosis is an intrinsic property of proteins encoded by the c-Myc locus [13,47,48]. However, naturally occurring truncations, mutations, or rearrangements in the c-Myc sequence rarely, if ever, adversely affect the mitogenic or pro-apoptotic activities of c-Myc proteins. This also applies—to some extent—to a viral analogue of c-Myc, v-Myc, which is a viral oncogene found in certain retroviruses. The genetic sequences of v-Myc and c-Myc share certain similarities, while the gene products demonstrate significant differences in functions. For instance, v-Myc is often more potent in promoting cancer than c-Myc due to differences in regulation and expression [38,39,40]. However, many human tumours exhibit genetic or epigenetic changes in c-Myc that disrupt the pathway underlying cell death to inhibit the pro-apoptotic activity of c-Myc [49,50,51,52]. However, it should be noted that the mechanism through which cell death is activated often remains intact, even in advanced malignancies, but is impeded so that proliferation is ultimately favoured [53,54,55,56].

The next section will provide a closer look at the locus structure of the central transcription regulator c-Myc. 

## 2. Structure of the c-Myc Locus

In humans, the production of more than one protein from a single genetic locus or mRNA is an event that occurs in some particular genes. One such case is the p16^INK4a^/p14^ARF^ locus, which encodes two distinct proteins with overlapping coding sequences but different reading frames [57,58]. Furthermore, it is well known that the human c-Myc locus, particularly the polycistronic c-Myc P0 transcript, can produce several distinct protein products.

**Figure 2:**
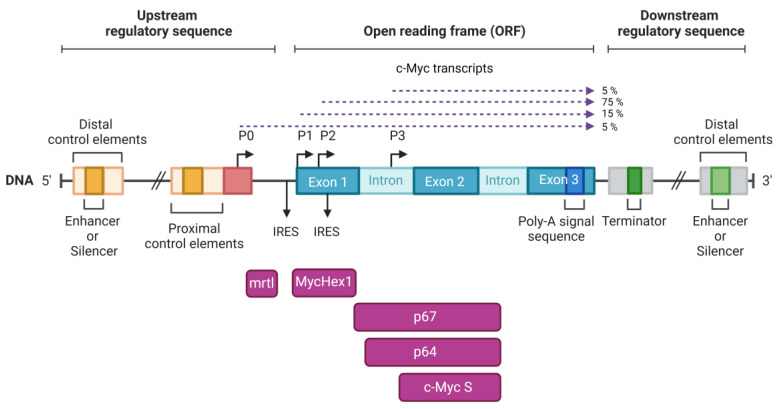
Diagram showing the structure of the c-Myc locus. The transcript starting from the P0 promoter encodes the mrtl and MycHex1 mRNAs, which are located upstream of the coding sequences for *p67* and *p64* mRNAs. The positions of the four transcription start sites (P0, P1, P2, P3) are indicated by bent arrows. Exons are indicated by dark blue rectangles and introns are indicated by light blue rectangles. Transcription rates initiated from four promoters are indicated by dashed dark purple arrows. Below the DNA coding sequence are all transcripts of the c-Myc locus indicated in light purple rectangles. Created with BioRender.com.

The human c-Myc locus, which is located on chromosome 8q24, has a complex structure (Figure 2). For instance, transcription can be initiated by binding to one of four alternative promoters (P0, P1, P2, and P3), the last of which is located between exons 1 and 2 of the gene [8,59,60,61]. The locus contains the coding sequences for two longer isoforms of the c-Myc protein, p67 (also termed c-Myc1) and p64 (referred to as c-Myc2), one truncated isoform of c-Myc S (p55), and the protein products of two ORFs, designated as mrtl and MycHex1. The sequences of mrtl and MycHex1 do not overlap with the c-Myc sequences, except for a minor overlap of 19 bp between the C-terminal sequence of MycHex1 and the N-terminal sequence of c-Myc p67, which are not in the same reading frame (Figure 2). P1 and P2 are the two most commonly used promoters, contributing to approximately 90% of the c-Myc transcripts in cells [62]. Translation of c-Myc mRNA can be initiated at one of two different initiation codons (CUG or AUG), leading to the synthesis of two protein isoforms (p64 and p67) [63]. p64 Myc is the predominant gene product, and most likely responsible for the oncogenic properties of the c-Myc locus [64]. In comparison to p64, the N-terminus of p67 Myc contains 14 additional amino acids and appears to have strong tumour suppressor properties. Thus, the p64:p67 ratio has a large influence on cell response [65].

## 3. Two Main c-Myc Isoforms: p64 and p67

The two major isoforms of c-Myc, p64 and p67, have been found in all vertebrate species studied to date [63]. In mammalian and avian cells, these two proteins are produced by the alternative initiation of translation at distinct in-frame codons, namely, the AUG codon for p64 and the CUG codon for p67 [63]. The evolutionary conservation of this c-Myc locus expression pattern over 400 million years suggests that multiple isoforms may play an essential role in c-Myc function. Translation of the p67 protein begins at the CUG codon, and thus results in an amino-terminal extension of 14 amino acids relative to the p64 protein [63]. Several lines of evidence suggest that the p67 protein is involved in cell growth and tumourigenesis. For instance, disruption of p67 synthesis has been observed in many Burkitt’s lymphomas [25,63]. In addition, the two forms of the c-Myc protein are differentially expressed during cell growth. When cell confluence increases, the p67 isoform predominates. This suggests that the p67 protein plays a role in growth inhibition, whereas the p64 and v-Myc proteins have been shown to stimulate growth. 

Many of the c-Myc gene rearrangements observed across various cancer types have been found to involve changes in exon 1. These often include complete deletion of exon 1 in the case of chromosomal translocations and retroviral translocations, along with minor deletions, point mutations, and proviral insertions in intron 1 [66]. The prevailing interpretation for this dynamic is that these changes only affect the regulation of c-Myc expression as the region encoding the protein with the AUG initiation codon in exon 2 is conserved. The first exon of c-Myc contains regions and regulatory elements that are likely important in controlling expression. These include sites for the addition of a methylguanosine cap to c-Myc mRNA at the 5′ end of exon 1 and a region that controls the elongation of nascent mRNA transcripts, as well as a possible enhancer element near the 3′ end of exon 1 [60,67,68,69,70,71]. Thus, removal of the first exon—which occurs in a series of rearrangements of the c-Myc locus—alters the c-Myc promoter structure and halts the expression of isoform p67, which is implicated in growth inhibition. Therefore, removal of the first exon of c-Myc likely leads to deregulation of the cell cycle and metabolism.

There also appear to be functional differences between the p64 and p67 proteins. For instance, p67 is a potent and specific transactivator of the enhancer element EFII via the C/EBP binding site (CCAAT-enhancer-binding protein, TTATGCAAT sequence). The C/EBP family consists of six related transcription factors which share a basic leucine zipper domain and are simultaneously classified as tumour suppressors, proto-oncogenes, and regulators of differentiation [72]. This transactivation has been observed in numerous cell types and species.

**Figure 3:**
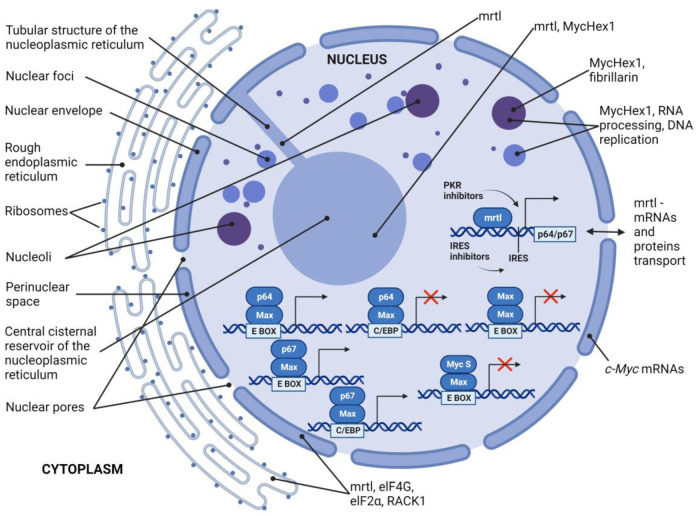
Schematic representation of the interaction and localisation of c-Myc isoforms. p64/Max, p67/Max, Myc S/Max, and Max/Max transcriptional activities on E-box and C/EBP responsive elements are depicted in the lower part of the figure, whereas localisation and function of mrtl and MycHex1 are shown in the upper part of the figure. Created with BioRender.com.

In contrast to the strong transactivation capacity of p67, the p64 c-Myc isoform either fails to transactivate the EFII enhancer element or represses EFII-driven transcription (Figure 3). In addition, there is evidence that v-Myc proteins also significantly repress transcription through interactions with the EFII enhancer element [65]. Both p64 and p67 proteins could transactivate via the canonical EMS sequence. Since both isoforms have the same C-terminal domain, the opposing effects of these proteins on EFII-driven transcription are most likely due to differences in N-terminal domains [27]. A possible explanation is that the amino terminal extension of 14 amino acid residues in p67 causes an overall conformational change in the N-terminal region, which contains the transactivation domain. These structural variations between p64 and p67 may result in unique interactions with transcriptional complexes based on specific DNA binding sites [65,73].

In addition to transcriptional activation, c-Myc has several distinct molecular functions, including transcriptional repression and direct modulation of DNA synthesis [23,74]. The N-terminal region of c-Myc is essential for the transcriptional repression of the cyclin D1 promoter [74]. The finding that p64 and p67 differentially transactivate the binding site for C/EBP yet can activate transcription of the EMS sequence suggests both distinct and overlapping functions for these two proteins. p67-regulated transcription of the C/EBP sequence indicates that the intracellular proportions of p64, p67, and C/EBP family members may determine the overall transcription rates of genes containing this sequence. Research conducted by Freytag and Geddes in 1992 highlighted that C/EBP and p64 proteins have contrasting roles in regulating adipogenesis [75]. Furthermore, there is evidence that an increase in p67 synthesis appears to be driven by methionine availability in the growth medium [76]. Thus, modulation of p67 levels may represent an early cell response to adapting to growth under nutrient deprivation. The different, and sometimes opposing, manners in which the two c-Myc proteins regulate the transcription may also apply to varying roles in the regulation of cellular metabolism.

Prior research has elucidated one of the cellular signalling mechanisms that regulates the intracellular balance of p64 and p67 levels. For instance, PKR (or EIF2AK2, eukaryotic translation initiation factor 2-alpha kinase 2) has been shown to enhance c-Myc transcription via interactions with NF-κB (Nuclear Factor Kappa-light-chain-enhancer of Activated B Cells) and STAT (Signal Transducer and Activator of Transcription). PKR activity also significantly influences c-Myc mRNA stability, translation, and subsequent protein stability [77]. Treating cells with a PKR inhibitor or performing siRNA-mediated knock-down of PKR results in heightened intracellular levels of p67. In contrast, PKR overexpression increases intracellular levels of p64 (Figure 3). This event is highly relevant to tumourigenesis, as the balance between p64 and p67 significantly impacts various cellular responses, such as proliferation, cell cycle arrest, and apoptosis. Interestingly, under normal growth conditions, PKR overexpression enhances cell growth, whereas the siRNA-mediated knock-down of PKR, or treatment with a PKR inhibitor, results in cell cycle arrest [77]. 

C/EBP overexpression also exerts a growth inhibitory effect [78]. However, since p64 and p67 proteins transactivate expression through EMS sequences, they may share some biological functions. It is likely that the disruption of p67 protein synthesis by genetic mutation or rearrangement, as is the case in Burkitt’s lymphoma, causes cells to lose the growth inhibitory response under nutrient depletion, which could contribute to oncogenesis.

## 4. The Third Isoform c-Myc S

In addition to p64 and p67, human, mouse, and avian cells also express smaller c-Myc proteins. These truncated proteins, termed c-Myc S, are produced by leaky scanning at conserved AUG codons downstream of the initiation sites for p64 in exon 2 of c-Myc (Figure 2) [79]. c-Myc S lacks most of the N-terminal transactivation domain present in p64 and p67 but retains the C-terminal dimerisation and DNA-binding domains (Figure 1). Like p64 and p67, the c-Myc S proteins are localised to the nucleus, can be phosphorylated, and are relatively unstable. Significant levels of c-Myc S, approaching those of p64 and p67, have been transiently observed during the rapid growth phase of several different cell types [79,80]. The c-Myc S proteins never activate the transcription of certain genes but are able to inhibit p64 and p67, which suggests a dominant-negative inhibitory function [80] (Figure 3). These smaller c-Myc proteins are obviously not expected to function as p64 and p67, while the finding that tumours express high levels of c-Myc S during the rapid cell growth phases suggests that these proteins do not impede the proliferative effects of p64 and p67. As such, although c-Myc S is characterised by the loss of most of the transcriptional activation domain found in both p64 and p67, it is a promoter of cell proliferation [79].

## 5. MycHex1 and mrtl

Evidence of an internal ribosomal entry site (IRES) designed solely for the translation of MycHex1 mRNA has sparked interest in deciphering the physiological roles of c-Myc P0 mRNA and the protein MycHex1 (Figure 2) [62]. Both mrtl and MycHex1 are found only in primates, in contrast to c-Myc, which is conserved across all vertebrates. mrtl and MycHex1 are relatively basic proteins, with pI values of 8.65 and 11.87, respectively. Full-length mrtl has 114 amino acids (12.5 kDa) and is rich in arginine. The N-terminal region, which is highly hydrophobic, is thought to be the only transmembrane domain (Figure 1) [40]. The hydrophobic region is interrupted by a series of charged amino acids (RSER). Another, slightly smaller isoform of mrtl exists, designated as mrtx (98 amino acids, 10.8 kDa), and lacks most of the transmembrane domain. Two myristoylation sites in the central region of the protein could further facilitate the membrane association of mrtl. The C-terminal sequence contains several examples of alternation between positively- and negatively-charged residues, and shows considerable homology to several RNA-binding proteins [40]. It is likely that this region serves as an interaction domain with other proteins [81]. Moreover, there are four sites at which serine residues can be phosphorylated (consensus substrates for protein kinase C, casein kinase II, protein kinase A, and protein kinase G), with two located in the middle of charged residues within the C-terminal domain [40].

Regulation of c-Myc expression at the translational level is also important to normal cell functioning [76,82,83]. The transcription of mrtl in cis from c-Myc mRNA places mrtl near regulatory sequences and controls the efficiency of c-Myc translation (Figure 2). The primary determinant of c-Myc translational regulation is the IRES sequence, which is located in the 5′ UTR between the coding sequences of mrtl and c-Myc (Figure 2) [84,85]. As such, it is possible that mrtl regulates c-Myc translation through modulation of IRES activity. From a genetic perspective, mrtl and c-Myc are very closely linked, so gene amplification or chromosomal translocations involving c-Myc will often affect the mrtl coding sequence [40]. Given this relationship between mrtl and c-Myc, it is plausible that mrtl may contribute to the role that the c-Myc locus plays in oncogenesis. 

Within the cell, mrtl is mainly found in the nuclear envelope, endoplasmic reticulum (ER), and tubular and cisternal structures of the nucleoplasmic reticulum (NR) [40] (Figure 3). Because the nuclear envelope and rough ER are studded with ribosomes, it is possible that mrtl is in close proximity to the translational apparatus. Thus, mrtl could be involved in the regulation of translation. There is already empirical evidence for this, as mrtl was found to be associated with the translation initiation factors elF4G (Eukaryotic Translation Initiation Factor 4 G) and elF2α (Eukaryotic Translation Initiation Factor 2α), as well as the integral 40S ribosomal protein RACK1 (Receptor For Activated C Kinase 1) [40].

In contrast, MycHex1 is present in nuclear foci, and only colocalised with mrtl at a single nuclear site, referred to as the central cisternal reservoir of the nucleoplasmic reticulum [86] (Figure 3). Prior research has shown that MycHex1 and fibrillarin shares their position at several discrete nuclear foci labelled with anti-BrdU antibody [86]. Fibrillarin is a ribonucleoprotein and nucleolar marker (snRNP) involved in ribosomal RNA processing [87]. Findings that BrdU is incorporated into DNA suggest that the nuclear loci at which MycHex1 is present may represent sites of DNA replication. As a highly basic protein, MycHex1 can associate with either DNA or RNA to facilitate replication or RNA processing, respectively [86]. 

Co-immunoprecipitation assays have revealed that endogenous mrtl and MycHex1 interact with RACK1, c-Myc, fibrillarin, coilin, and even with each other [86]. This indicates that both proteins may bind to a wide array of partner molecules within the nucleus and cytoplasm. Given the structural characteristics of mrtl and MycHex1, it is plausible that these proteins serve to anchor essential protein assemblies by targeting protein regions that include amino acid residues with alternating charges. Notably, the abundance of arginine and serine in both proteins is indicative of similarity to numerous RNA-binding proteins [86]. 

The cell nucleus has a sophisticated structure and houses several unique parts, such as nuclear bodies, nucleoli, Cajal bodies, nuclear speckles, paraspeckles, PML bodies (promyelocytic leukaemia), and Polycomb bodies. Notably, these nuclear entities lack a defining membrane, which enables the seamless interchanging of contents with the adjacent nucleoplasm [88]. MycHex1 might play a pivotal role in the formation and fortification of certain nuclear bodies. The ability of MycHex1 to undergo homo-oligomerisation, when combined with the co-immunoprecipitation findings that this protein has several potential binding partners, aligns well with this hypothesised role [89]. The nucleoplasmic reticulum comprises a series of membranous tubules within the nucleus to form the central cisternal reservoir [90,91,92,93,94]. This structure involves folds of the nuclear envelope and has a similar composition as the cytoplasm. The numerous folds in this nuclear structure significantly increase the surface area and enhance contact between the nucleus and cytoplasm. The distribution of mrtl across the nuclear envelope and nucleoplasmic reticulum alludes to a mechanism in which mrtl mediates the transport of certain molecules between the nucleus and cytoplasm [40] (Figure 3).

It is widely recognised that proteins which will be fully or partially integrated into the cell membrane will first be processed at the endoplasmic reticulum (ER) membrane via interactions between a signal peptide and a signal recognition particle. Analogously, proteins like c-Myc, which are localised to the nucleus, might undergo a similar co-translational transfer so that the synthesised protein is transported across the nuclear membrane into the nucleoplasm. c-Myc mRNA is predominantly found in the perinuclear area (Figure 3). The distinct presence of mrtl at the nuclear membrane and nucleoplasmic reticulum, coupled with structural similarities to ATP-binding cassette (ABC) transport proteins, hints at a potential role in translocating nascent c-Myc into the nucleus [95]. In addition to specific regulation of c-Myc translation, mrtl might also influence the translational efficiency of other mRNAs. Indications of a broader cellular role for mrtl include extensive presence across the endoplasmic and nucleoplasmic reticulum, consistent accumulation in cells, and deep integration within cellular structures. As such, mrtl could facilitate interactions between mRNA, translational machinery, and the intracellular membrane network (Figure 3). As mrtl is positioned at a junction of the cytoplasm and nucleus, it might play a crucial role in synchronising the movement of mRNAs and nascent proteins between the cytoplasm and nucleus [96]. 

## 6. Targeting c-Myc in Cancer

c-Myc is implicated in various cancers and other diseases, including but not limited to lymphomas, breast cancer, lung cancer, colorectal cancer, and prostate cancer. The role and significance of c-Myc may vary across different cancer types [9]. High levels of c-Myc expression in certain cancers has been correlated with poor prognosis. It is often associated with more aggressive tumour behaviour, increased likelihood of metastasis, and resistance to treatment. Changes in c-Myc expression levels during the course of treatment may serve as an indicator of treatment response. Monitoring c-Myc levels can help assess the effectiveness of therapies and guide treatment decisions [9].

The c-Myc transcription factor has emerged as a significant target for therapeutic intervention, particularly in the context of cancer and other diseases as well [97,98]. Given its crucial role in promoting tumorigenesis and its frequent dysregulation in various malignancies, researchers have explored two primary strategies for inhibiting c-Myc: direct inhibition of its activity and indirect approaches that modulate its expression or stability [99].

Direct inhibition involves the development of therapeutics designed to disrupt its transcriptional activity, modulate its interactions with co-factors, or cause G quadruplex stabilization in its promotor. This approach, although challenging due to the lack of well-defined binding pockets on c-Myc, holds promise for precise targeting of the oncoprotein. Further strategies in this category include antisense oligonucleotides (ASOs), which target and degrade c-Myc mRNA and miniproteins designed to block its DNA binding domain [98]. 

On the other hand, indirect inhibition focuses on manipulating pathways upstream or downstream of c-Myc. Strategies in this category also include modulation of c-Myc degradation and protein stability mostly via post-translational modifications [20,100]. 

A list of compounds belonging to direct and indirect c-Myc inhibitors is summarized in Table 1.

Regarding the direct influence of p64 and p67 isoforms ratio by small molecules, an extensive high throughput screening of 135,000 compounds was performed by Vaklavas and colleagues [101]. Among them, an inhibitor of IRES-mediated translation was identified. The structure and activity of this hit was greatly improved leading to the development of cpd_P. This cpd_P is causing complete loss of clonogenic survival, massive cell death, terminal differentiation, and death of putative tumour stem cells [102].

To discover another IRES modulator inhibiting c-Myc translation, named J007, a library of 145,000 compounds had to be tested. The effort certainly paid off as J007 inhibits proliferation of multiple myeloma cell lines and tumour growth in vivo [103]. Furthermore, it induces cell death in glioblastoma resistant to mechanistic targeting of rapamycin (mTOR) inhibition when J007 and the PP242 (mTOR inhibitor) are simultaneously applied [104]. The effect of J007 on the expression of p64 and p67 isoforms remains to be elucidated.

## 7. Discussion and Summary

The primary objective of this literature review was to comprehensively present the reasons why the c-Myc gene plays such a crucial role in determining cell fate. We have explained how expression of this oncogene can produce five different proteins, each with unique characteristics and functions. These proteins have distinct structures, are localised to different cell compartments, and exert unique roles (Table 2). This genomic arrangement highlights the multifaceted nature of c-Myc expression and functions.

Throughout this article, we have discussed the unique features of the c-Myc gene within the human genome and focused on two well-established yet controversial roles: stimulating cell cycle progression and promoting growth arrest and apoptosis. Meticulous research into these roles can provide insight as to why the expression of five distinct c-Myc protein isoforms is necessary for regulating normal c-Myc function during cell growth and arrest. The complex regulatory landscape surrounding c-Myc-mediated cellular dynamics is emphasised by the different mechanisms governing the synthesis of various c-Myc isoforms, demonstrating varying abilities to activate and regulate transcription.

Previous research has shown that disruptions in the balance between two specific c-Myc protein isoforms, p64 and p67, are often observed in cancer cell lines with deregulated c-Myc activity. These imbalances in isoform proportions may directly contribute to the loss of control over cell growth, which is a common feature of tumourigenesis. Understanding the molecular intricacies that govern how c-Myc is involved in cell cycle regulation is crucial due to frequent dysregulation in human cancers. The relationships between various c-Myc protein isoforms, particularly the specific impacts on cell cycle control and arrest, open new possibilities for therapeutic interventions. The definitive understanding of the complex interplay between c-Myc, apoptosis, and cell cycle progression could significantly address a wide range of malignancies.

## 8. Conclusions

In conclusion, ongoing efforts to understand the regulatory mechanisms underlying c-Myc-mediated cell cycle control offer promising prospects for innovative therapeutic strategies against c-Myc-related cancers [98]. However, it is crucial to consider the dual role of c-Myc, i.e., promotion of apoptosis and support of cell survival and differentiation, when designing interventions. Further uncovering the complexities of c-Myc function will allow researchers to gain a deeper understanding of how this transcription factor contributes to cancer biology. We believe that the most effective therapeutic potential lies in restoring the balance between p64 and p67, as demonstrated by studies that included the inhibition of PKR and IRES-mediated translation [77,101,102,105,106]. The optimal utilisation of this regulatory mechanism will require additional research into other proteins that may influence this balance. Compounds that modulate PKR activity, IRES-mediated translation, and other relevant proteins could be crucial to targeted treatments for aggressive malignancies. Expanding the range of c-Myc modulators will be pivotal to the identification of alternative strategies in cases of resistance and enable more personalised treatment options for tumours of different origins.

## Figures and Tables

**Table 1:** A list of compounds and drug candidates directly and indirectly inhibiting c-Myc.

**Indirect c-Myc inhibition**	BET family inhibitors	JQ1, Birabresib (OTX015, MK-8628), Molibresib (GSK525762), RO6870810 (RG6146, TEN-0), FT-1101 (CC-95775), ZEN-3694, BMS-986158, AZD5153, BI894999, CPI-0610, GSK2820151, INCB057643, INCB054329 and GS-5829, TEN-010, ABBV-075, PROTACs ARV-771, and ARV-825
	BCR inhibition	Ibrutinib, ARQ531
	eIF4A inhibition	Silvestrol
**Indirect c-Myc inhibition**	PI3K inhibition	Idelalisib, TGR-1202, Fimepinostat (CUDC-907), BR101801
	CDK inhibition	Dinacyclib, TG02, KB-0742,THZ1 and THZ2, aminopyrimidines, triazane derivatives, carbamoyl sulfoximide, 4-(4-fluoro-2-methoxyphenyl)-N-1,3,5-triazin-2-amine
	PIM1 inhibition	AZD1208, SGI-1776, TP-3654 (SGI-9481), MEN1703, PIM447
	PIN1 inhibition	KPT-6566, Retinoid ATRA, BJP-06-005-3, Sulfopin, PIM447, SEL24 (MEN1703)
	PP2A modulation	DT-061, FTY720, OP449, Perphenazine, LB-100
	SKP2 inhibition	SZL-P1-41, FKA, Dioscin, SKPin C1
	USP7 inhibition	P22077, XL177A, GNE-6640, GNE6776, FT671
	JAK2/STAT3 inhibition	MTAP-26, and MTAP-27, WP1066, WP1130, and WP1129
	NF-ĸB inhibition	Guggulsterone
	Src kinase inhibition	Saracatinib
	FBXW7 activation	Oridonin, HAO472
	Aurora-A inhibition	Alisertib (MLN8054, MLN8237), CD532
	Aurora-B inhibition	AZD1152
	PLK-1 inhibition	BI6727
	HUWE1 inhibition	BI8622 a BI8626
	HDAC inhibition	Entinostat, Tucidinostat, CUDC-907
**Direct c-Myc inhibition**	G quadruplex stabilisation	CX-3543, APTO-253, IZCZ-3, cationic porphyrins (TMPyP4), quarfloxin, DM039, ruthenium complexes (Se2Py3, Se2SAP)
	Antisense oligonucleotides	AVI- 4126, MYC-ASO, INX-3280, INX-6295
	Miniproteins and protein domains	OmoMYCs (OMO-103, OMO-1, FPPa-OmoMYC), Bac- ELP-H1, PNDD1, ME47, Mad, alfa-helix peptide H1
	Myc/Max interaction disruption	ME47, EN4, 3jc48-3, pyrazolo [1,5-a]-pyrimidines (MYCro1, MYCro2 a Mycro3), KJ-Pyr-9 (Kröhnke pyridine), MYCMI-6, MYCMI-7, MYCi975, MYCi361, KSI-3716, MYRA-A, MI1-PD, KI-MS2-008, quinolone derivatives (KSI-1449, KSI-2302, and KSI-3716), substituted pyrazole compounds (NUCC-0176242, and NUCC-0176248), IIA6B17, 10058-F4, 10074-G5, JY-3-094, JKY-2-169, SaJM589
	Max/Max homodimers sabilization	KI-MS2-008, NSC13728

Note: BET family (Bromodomain and Extra-terminal Domain), BCR (The Breakpoint Cluster Region Protein), eIF4A (Eukaryotic Translation Initiation Factor 4A), PI3K (Phosphatidylinositol 3-Kinase), CDK (Cyclin-dependent kinase), PIM1 (Pim-1 Proto-Oncogene, Serine/Threonine Kinase), PIN1 (Peptidylprolyl Cis/Trans Isomerase, NIMA-Interacting 1), PP2A (Protein phosphatase 2A), SKP2 ((S-Phase Kinase Associated Protein 2), USP7 (Ubiquitin Specific Peptidase 7), JAK2 (Janus kinase 2), STAT3 (Signal Transducer And Activator Of Transcription 3), NF-ĸB (Nuclear Factor Kappa B), Src (SRC Proto-Oncogene, Non-Receptor Tyrosine Kinase), FBXW7 (F-Box And WD Repeat Domain Containing 7), PLK-1 (Polo Like Kinase 1), HUWE1 (HECT, UBA And WWE Domain Containing E3 Ubiquitin Protein Ligase 1), HDAC (Histone Deacetylase), Max (MYC Associated Factor X), Myc (MYC Proto-Oncogene, BHLH Transcription Factor).

**Table 2:** Summary and comparison of the most important findings on all isoforms of c-Myc.

	p64 Myc (c-Myc2)	p67 Myc (c-Myc1)	mrtl	MycHex1	c-Myc S
**Structure**	well known	contains additional 14 amino acids at its N terminus compared to p64 Myc	N-terminal region single transmembrane domain, C-terminal sequence interaction domain with homology to RNA-binding proteins	highly basic protein, capable of homo-oligomerization	c-Myc S lacks the N-terminal transactivation domain
**Expression**	predominant gene product of the c-Myc locus	lost in many tumours	unknown	IRES facilitates translation of the MycHex1	higher levels of c-Myc S have been transiently observed during the rapid growth phase of several cell types
	**p64 Myc** **(c-Myc2)**	**p67 Myc** **(c-Myc1)**	**mrtl**	**MycHex1**	**c-Myc S**
**Function**	oncogenic properties, p64 c-Myc isoform transactivates via the canonical EMS sequence and fails to transactivate the EFII enhancer element via the C/EBP binding site	growth inhibitory properties, p67 is a potent and specific transactivator of the enhancer element EFII via the C/EBP binding site and also transactivates via the canonical EMS sequence, mediates growth inhibitory response under nutrient depletion or contact inhibition	regulates c-Myc translation and localization to the nucleus, contributes to the role of the c-Myc locus in oncogenesis (IRES), might be part of a complex which regulates the translation, localization, or processing of mRNA	possibly involved in replication, RNA processing, and formation of nuclear bodies	c-Myc S protein lacks transactivation capacity, but it is able to inhibit p64 and p67, which suggests a dominant-negative inhibitory function
**Onthology**	conserved in chimpanzee, Rhesus monkey, dog, cow, mouse, rat, chicken, zebrafish, and frog		mrtl and MycHex1 are found only in primates		human, mouse, and avian cells
**Subcellular localisation**	mainly nucleus and cytoplasm	mainly nucleus and cytoplasm	nuclear envelope, ER, tubular and cisternal structures of the NR	colocalizes with fibrillarin	mainly nucleus and cytoplasm
**Additional information**	stoichiometric balance between p64 and p67 is important for cellular metabolism regulation and proliferation		colocalize in the central cisternal reservoir of the nucleoplasmic reticulum		

## Data Availability

Not applicable.
